# Optimization of Virtual Shack-Hartmann Wavefront Sensing

**DOI:** 10.3390/s21144698

**Published:** 2021-07-09

**Authors:** Xian Yue, Yaliang Yang, Fei Xiao, Hao Dai, Chao Geng, Yudong Zhang

**Affiliations:** 1 Key Laboratory of Adaptive Optics, Chinese Academy of Sciences, Chengdu 610209, China; yuexian17@mails.ucas.ac.cn (X.Y.); ustcxiaofei@163.com (F.X.); daihao20@mails.ucas.ac.cn (H.D.); gengchao@ioe.ac.cn (C.G.); ydzhang@ioe.ac.cn (Y.Z.); 2 Institute of Optics and Electronics, Chinese Academy of Sciences, Chengdu 610209, China; 3 University of Chinese Academy of Sciences, Beijing 100049, China

**Keywords:** wavefront sensing, aberration measurement, Shack–Hartmann wavefront sensing, digital wavefront processing, parameter optimization

## Abstract

Virtual Shack–Hartmann wavefront sensing (vSHWS) can flexibly adjust parameters to meet different requirements without changing the system, and it is a promising means for aberration measurement. However, how to optimize its parameters to achieve the best performance is rarely discussed. In this work, the data processing procedure and methods of vSHWS were demonstrated by using a set of normal human ocular aberrations as an example. The shapes (round and square) of a virtual lenslet, the zero-padding of the sub-aperture electric field, sub-aperture number, as well as the sequences (before and after diffraction calculation), algorithms, and interval of data interpolation, were analyzed to find the optimal configuration. The effect of the above optimizations on its anti-noise performance was also studied. The Zernike coefficient errors and the root mean square of the wavefront error between the reconstructed and preset wavefronts were used for performance evaluation. The performance of the optimized vSHWS could be significantly improved compared to that of a non-optimized one, which was also verified with 20 sets of clinical human ocular aberrations. This work makes the vSHWS’s implementation clearer, and the optimization methods and the obtained results are of great significance for its applications.

## 1. Introduction

Shack–Hartmann wavefront sensing (SHWS) [1] is currently the main means of wavefront aberration measurements in astronomy [2], high-energy laser [3], retinal imaging [4], optical communication [5], and optical testing [6] due to its simple principle. SHWS consists of a lenslet array (LA) and a 2D detector. Each lenslet is a sub-aperture, and the incident beam is divided into multiple individual sub-beams and is then focused on the 2D detector by the LA. The focused spots of a collimated incident beam with a planar wavefront are defined as the reference spots without wavefront aberrations. By measuring the displacement Δs of the focused spot centroid from the reference spot centroid, the slope *θ* of a sub-wavefront can be obtained by *θ* = Δs/*F*, where *F* is the focal length of lenslet. After obtaining all the sub-wavefront slopes, the entire wavefront at the pupil can be calculated by using a reconstruction algorithm [7]. A one-shot signal capturing in a time on the order of a millisecond to obtain the entire wavefront [8] allows for real-time aberration measurements. Although the parameters of SHWS can be carefully designed to meet a determinate application requirement, there are still some limitations in practice: First, due to the use of LA, the system and alignment are more complex, and larger non-common-path errors between wavefront sensing and science paths may be introduced. Second, it is difficult to change the LA to meet different aberration measurements once it has been produced and mounted in system because the sub-aperture number is related to the maximum order of the Zernike aberrations that can be measured reliably [9]. Lastly, due to the low numerical aperture of the lenslets, SHWS is not sensitive to the aberration change along the depth, and it also cannot resist the stray reflections from the interfaces of optics and specimen [10] in the case of biomedical imaging.

Feierabend et al. proposed a coherence-gated virtual SHWS (CG-vSHWS) in 2004 [11], which was a combination of coherence-gated wavefront sensing (CGWS) and virtual SHWS (vSHWS). CGWS is based on a low-coherence interferometer and can only extract the backscattered light from a short depth range within a sample with a short coherence gate [12]. Interferometric phase measurement has a π-ambiguity in the arctangent function, and phase unwrapping is needed for CGWS. However, phase singularities occur at the points where the amplitudes of interference signals go to zero, thus decreasing the phase unwrapping accuracy [13]. Both conventional and advanced phase unwrapping algorithms are not satisfactory for the wrapped phase with a strong noise and a lot of singularities [14]. This problem can be avoided when using vSHWS because it processes a measured electric field instead of a wrapped phase.

After the electric field at the pupil is measured by CGWS, vSHWS can be used to obtain the wavefront aberrations. The measured electric field is first digitally divided into multiple independent sub-areas, and then a digital propagation process is performed to each sub-area to generate a pseudo spot at the geometric focal plane [15] of a virtual LA (v-LA). The wavefront is finally obtained by using the slopes of all sub-aperture wavefronts with the reconstruction algorithm. In addition to the advantages inherited from SHWS, vSHWS has other advantages: (1) Since a digital method instead of a real (physical) LA is used to divide the electric field, it is easy to change the sub-aperture number according to the Zernike modes to be calculated [9,16]. (2) Without the focusing effect of the LA, the light intensity received by the 2D detector is more uniform, which is favorable to detect weak signal and improve wavefront sensing sensitivity. (3) A real LA is not required, which can simplify the system and alignment, as well as reduce the non-common-path error. (4) The errors caused by focal shift [15] and non-uniform illumination [17] in SHWS do not present. (5) The procedure, methods, and parameters of data processing can be flexibly adjusted to achieve a high performance via optimization. Therefore, vSHWS is a more promising means for accurate phase unwrapping and wavefront sensing in some applications, such as adaptive optics optical coherence tomography for in vivo retinal imaging.

As a relatively new technology, there are few reports on analyzing the characteristics of vSHWS [12] and using it for wavefront sensing [18,19,20,21]. Data processing methods and parameter selections are all related to the performance of wavefront sensing. Wang et al. demonstrated that the performance of vSHWS could be improved by performing the zero-padding of a sub-aperture electric field and by optimizing the lenslet size [22], but how to find the optimal configuration has not been described in detail. In addition, the effects of other factors on the performance of vSHWS, such as the shape of virtual lenslet, the sub-aperture number, and data interpolation, etc., have never been studied.

Starting from the principle of vSHWS, in this work the data processing procedure was illustrated by applying it to ophthalmology and using a set of human ocular aberrations as an example. The effects of all factors on the performance of vSHWS, including the shape of the virtual lenslet, the zero-padding of a sub-aperture electric field, the sub-aperture number as well as the sequences, algorithms, and intervals of data interpolation were studied, and then an optimal configuration was obtained. The wavefront sensing and the anti-noise performances of vSHWS with and without optimizations were compared. The purposes of this work are to further clarify the working procedure of vSHWS and to provide the optimization methods and conclusions for its applications.

## 2. Methods

### 2.1. Implementation of vSHWS

The complex electric field *E* to be analyzed needs to be obtained first by using any method, such as a four-step phase shifting interferometry. In detail, four interference images *I*_1_, *I*_2_, *I*_3_ and *I*_4_ can be obtain, and *E* is given by [11]:

(1) $$\begin{array}{ll} E & =\left(I_{1}-I_{3}\right)+i\left(I_{4}-I_{2}\right)\\ & =const\cdot \left(\cos\Delta \varphi +i\sin\Delta \varphi \right)\\ & =const\cdot \exp\left[i\Delta \varphi \right] \end{array},$$

where *const* is a constant, Δ*φ* is the phase difference between the sample and reference arms, and *i* is the imaginary sign. The working procedure of vSHWS is briefly described as follows: Generate a v-LA matrix, then multiply *E* by the v-LA matrix to obtain an amplitude-filtered *E*. Divide the amplitude-filtered *E* into multiple sub-areas corresponding to virtual sub-apertures, and then calculate the Fraunhofer diffraction of each sub-area to obtain diffracted spot at the focal plane of the v-LA. Calculate the centroid positions of all diffracted spots, and calculate the slopes of all sub-aperture wavefronts by using the displacements between the calculated and reference centroid positions. Finally, find the Zernike coefficients via least-squares fitting the slopes with the Zernike polynomials, and then reconstruct the wavefront by using the Zernike coefficients.

**Figure 1:**
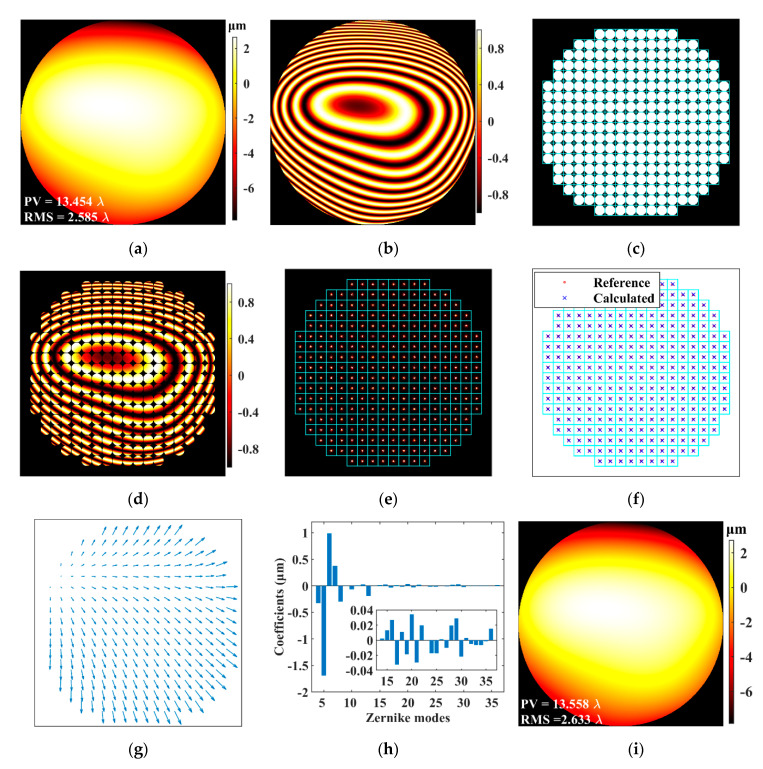
Main data processing steps and corresponding results of vSHWS. (**a**) Wavefront *W*_P_ to be measured; (**b**) real part of complex electric field *E* generated from (**a**); (**c**) virtual LA; (**d**) real part of amplitude-filtered *E*; (**e**) diffracted spots of the entire aperture; (**f**) calculated and reference centroid positions; (**g**) displacement vectors of centroids; (**h**) calculated Zernike coefficients; (**i**) reconstructed wavefront *W*_R_.

The following was performed by way of example to illustrate the working procedure of vSHWS in detail. The effective area detecting the interference images was assumed to 1200 × 1200 pixels, i.e., the wavefront *W*_P_ to be measured was sampled by 1200 × 1200 points, the pixel size of the 2D detector was 10 μm, the wavelength *λ* was 780 nm, and the focal length *F* of the virtual lenslet was 10 mm. All the values were within the ranges of commercial devices. According to Equation (1), the preset *E* to be processed can be generated by

(2) $$E=A\cdot \exp\left[i2\pi /\lambda \cdot W_{P}\right],$$

where the amplitude *A* is set to 1 in this study, *W*_P_ is the preset wavefront, and (2π/λ·*W*_P_) is equal to Δ*φ* in Equation (1). *W*_P_ was generated by using a set of Zernike coefficients of human ocular aberrations [23,24], which were statistical results obtained by measuring 200 healthy and normal eyes at a pupil diameter of 6 mm [25]. Figure 1a shows the *W*_P_ including from the 4th to 36th terms of the Zernike coefficients (i.e., 7-order aberrations usually need to be measured and corrected in adaptive optics ophthalmic imaging), and its peak-to-valley (PV) and root mean square (RMS) are 13.454 *λ* and 2.585 *λ*, respectively. The Zernike order used in this article is the standard arrangement recommended by the Optical Society of America [26]. The first three terms of the Zernike aberrations, including the piston, tip, and tilt, usually cannot be sensed and are thus excluded. The real part of the preset *E* is shown in Figure 1b. We then used vSHWS to process *E* to obtain the Zernike coefficients; a reconstructed wavefront *W*_R_ was obtained by using the obtained Zernike coefficients, and the performance of vSHWS could be evaluated by comparing *W*_R_ and *W*_P_.

*E* can be expressed by a matrix, and the shape of its effective area illuminated by the beam is usually a round. After digitally dividing the entire amplitude-filtered *E* into multiple sub-apertures, only the sub-apertures filled with the effective *E* signal can be reserved, called effective sub-apertures. According to the design principle of SHWS, the number of the effective sub-apertures should not be less than the number of the Zernike coefficients that need to be calculated reliably [16]. The shape of the virtual lenslet was designed to be round first. The arrangement of the sub-apertures was a 20 × 20 grid; a single sub-aperture was thus sampled by 60 × 60 points, and the number of the effective sub-apertures was 276. Figure 1c shows the arrangement of the sub-apertures, and each cyan grid represents a sub-aperture. In fact, it is a 2D transmittance function, where the transmittances are 1 at the white areas and 0 at the black areas. The obtained *E* matrix was multiplied by the transmittance matrix, and then the data in each sub-aperture were taken out separately to obtain the *E*_0_ matrix that is passed through a single virtual lenslet. Figure 1d shows the real part of the amplitude-filtered *E*.

The Fraunhofer diffraction [27] of the *E*_0_ in each sub-aperture was calculated separately to simulate the processes in SHWS, i.e., light propagating through an LA and then focused on a 2D detector by the LA. Taking the geometric focal length *F* [15] of the virtual lenslet as the observation distance of the Fraunhofer diffraction, the diffracted electric field *E*_F_ on the 2D detector is given by the following [27,28]:

(3) $$\begin{array}{ll} E_{F}(x_{F},y_{F}) & =\frac{\exp\left[ikF\right]}{i\lambda F}\cdot \exp\left[ik\frac{x_{F}^{2}+y_{F}^{2}}{2F}\right]\iint_{\Sigma }E_{0}(x_{0},y_{0})\cdot \exp\left[-ik\frac{x_{0}x_{F}+y_{0}y_{F}}{2F}\right]dx_{0}dy_{0}\\ & =\frac{\exp\left[ikF\right]}{i\lambda F}\cdot \exp\left[ik\frac{x_{F}^{2}+y_{F}^{2}}{2F}\right]\cdot \mathrm{FT}\left[E_{0}(x_{0},y_{0})\right] \end{array},$$

where (*x*_0_, *y*_0_) and (*x*_F_, *y*_F_) are the coordinates of the incident *E*_0_ and diffracted *E*_F_, respectively; wavenumber *k*=2π/*λ*; and FT[·] is Fourier transform. Therefore, the diffracted spot *I*_F_, i.e., the intensity of *E*_F_, is given by

(4) $$I_{F}\left(x_{F},y_{F}\right)=E_{F}\left(x_{F},y_{F}\right)\cdot \mathrm{Conj}\left[E_{F}\left(x_{F},y_{F}\right)\right],$$

where Conj[·] means conjugate calculation. The side length *L* of the diffracted spot *I*_F_ is given by the following [18]:

(5) $$L=N\lambda F/L_{0},$$

where *N* is the sample point number of *E*_0_, and *L*_0_ is the side length of *E*_0_ in a square shape and is 0.6 mm in this example.

**Figure 2:**
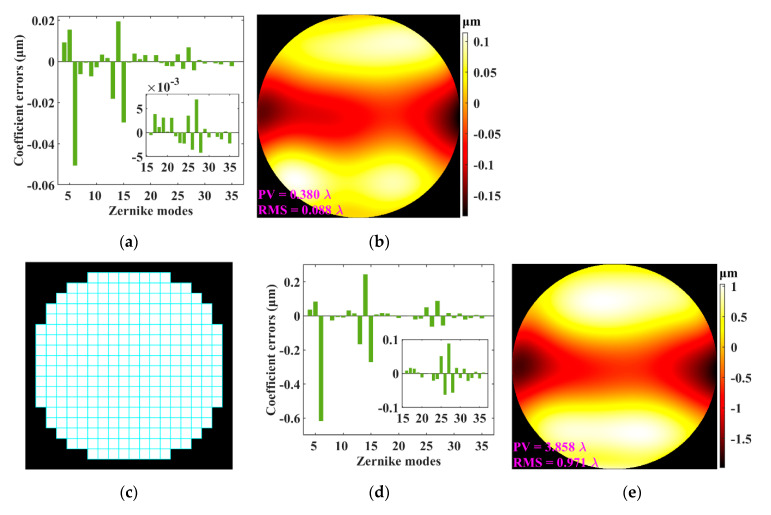
Results of vSHWS using round and square lenslets. (**a**) Zernike coefficient errors and (**b**) wavefront error when using round lenslets; (**c**) arrangement of square lenslets; (**d**) Zernike coefficient errors and (**e**) wavefront error when using square lenslets.

Figure 1e shows the diffracted spots of the entire aperture. The centroid position of each diffracted spot was calculated and then compared with the corresponding reference position to obtain the displacement vector of the centroid. Figure 1f shows the calculated and the reference centroid positions, and Figure 1g shows the displacement vectors of the centroids. The slope of each sub-aperture wavefront was obtained by using the displacement vector, and the Zernike coefficients shown as Figure 1h were calculated by using a Zernike modal reconstruction [7]. The wavefront *W*_R_ shown as Figure 1i was reconstructed by using the obtained Zernike coefficients, and its PV and RMS were 13.558 and 2.633 *λ*, respectively, close to those of *W*_P_ shown as Figure 1a. The Zernike coefficient errors and the wavefront error between *W*_P_ and *W*_R_ are shown in Figure 2a,b, respectively.

### 2.2. Optimization of vSHWS

Step by step, the parameters obtained from the previous optimizations were used as the preset parameters for the next optimization, until all optimizations were completed. For the 1200 × 1200 sample points of *W*_P_, it is convenient to use different sub-aperture sizes, such as 30 × 30 and 80 × 80 points, etc., because it can be divisible by more sizes. The changes of the Zernike coefficients under different parameters and the RMS of the wavefront error between *W*_R_ and *W*_P_ were used to evaluate the performance of the vSHWS.

There are mainly three shapes for the lenslet of a commercial LA: round (e.g., MLA300-14AR, Thorlabs Inc., Newton, NJ, USA), square (e.g., MLA150-7AR, Thorlabs), and hexagonal (e.g., AHP-Q-P1000-R3, AMS Inc., Ismaning, Germany). Accordingly, the shape of the virtual lenslet, i.e., the light-transmitting sub-aperture, also has three options. Although an LA with hexagonal lenslets has a higher fill factor, i.e., higher signal utilization efficiency (close to 100%), its shape dividing and data processing are more complicated. Therefore, only the v-LAs with round and square lenslets were investigated in this study. The v-LA with round virtual lenslets was investigated in Section 2.1, but that with square virtual lenslets should theoretically have a better performance due to its higher fill factor (also close to 100%). In order to verify whether this theoretical intuition was true, the shape of the virtual lenslets was changed to be square without changing other parameters, and then the processing procedure described in Section 2.1 was repeated to obtain the results. The Zernike coefficient errors and the RMSs of the wavefront errors obtained from the two simulations with the round and square virtual lenslets were compared to find the better one.

In SHWS, all sub-beams are focused on the 2D detector by the LA, simultaneously. If the slope of a local sub-wavefront is too large, the focused spot falls outside its sub-aperture area on the detector and forms overlapped spots and spot crossovers with the adjacent sub-apertures, which limits the dynamic range of SHWS. By contrast, in vSHWS, the data of each sub-aperture is processed individually, and thus the focused spot never overlaps with others. It can be seen from Equation (5) that *L* is a function of *N* and *L*_0_; if *N* and *L*_0_ are increased by the same times, *L* does not change, but the sampling frequency of the observation plane increases. Therefore, the performance of vSHWS can be improved by expanding *E*_0_, or more specifically, by zero-padding *E*_0_, i.e., padding zeros to the edge of *E*_0_ to increase *N*. The expansion ratio was increased from 1 (implying no padding), and the RMS plots of the wavefront errors varying with the expansion ratio were recorded to determine the optimal expansion ratio, which had a balanced performance and computation burden.

The size of the illuminated area on the image plane is a constant in a system, i.e., the sample point of the detected *E* is definite, and thus there is a negative correlation between the size and number of the sub-aperture. There is a trade-off between them: a larger sub-aperture size means that more light signals can be used for spot centroid calculation, facilitating accurate wavefront measurement; however, increasing the sub-aperture size reduces its number and accordingly decreases the spatial sampling rate of the wavefront, which limits the accuracy of wavefront sensing. Therefore, an appropriate sub-aperture number is important for both SHWS and vSHWS. Unlike SHWS, the sub-aperture number of vSHWS can be adjusted easily, which is beneficial for dynamically adjusting the performance of wavefront sensing. While maintaining 1200 × 1200 sample points of the preset *E*, the sub-aperture number was set to 10 × 10, 12 × 12, 15 × 15, 20 × 20, 24 × 24, 30 × 30, 40 × 40, and 60 × 60 to reconstruct *W*_P_ sequentially. The RMSs of the wavefront errors between *W*_R_ and *W*_P_ were calculated to find the optimal sub-aperture number.

The focused spot centroid can be calculated more accurately by using a 2D detector with a smaller pixel size, which can improve the sensitivity of wavefront sensing. However, a smaller size pixel is more sensitive to noise, which reduces the accuracy of wavefront sensing. The pixel size of a used 2D detector cannot be changed, and thus data interpolation may be a way to improve the performance of wavefront sensing. The data interpolation can be performed before or after the diffraction calculation (DC), i.e., performed on the sub-aperture *E*_0_ or the diffracted spot *I*_F_, and the interpolation algorithm and interval can also be flexibly changed. Therefore, two interpolation sequences (i.e., before and after the DC), commonly used three interpolation algorithms (i.e., linear, cubic, and spline), and different interpolation intervals were investigated so that we could observe their effects on the performance of vSHWS and subsequently find their optimal combination with the highest accuracy of wavefront sensing.

## 3. Results

### 3.1. Shape of Virtual Lenslet

Except for changing the shape of the virtual lenslets from round to square, as shown in Figure 2c, the same procedure and parameters as in Section 2.1 were used to reconstruct the preset wavefront *W*_P_, and the results are shown in Figure 2d,e. In comparing Figure 2a,d, it can be seen that the Zernike coefficient errors obtained by using the round lenslets are about an order of magnitude smaller than those obtained by using the square lenslets. Figure 2b,e shows the wavefront errors when using the round and the square lenslets, respectively; it can also be seen that the RMS (0.088 *λ*) when using the former is also an order of magnitude smaller than when using the latter (RMS = 0.971 *λ*). Therefore, the performance of vSHWS when using the round lenslets is significantly better than when using the square lenslets, which is contrary to the expectation that the latter with a higher fill factor should have a better performance. The reason might be that in order to facilitate the calculation, the round transmitting area of each round lenslet was zero-padded to form a matrix, which was essentially a pre-zero-padding of *E*_0_ and thus could improve the performance, but there was no such pre-zero-padding for each square lenslet. This assumption would be confirmed in Section 3.2.

### 3.2. Zero-Padding of Sub-Aperture Electric Field

**Figure 3:**
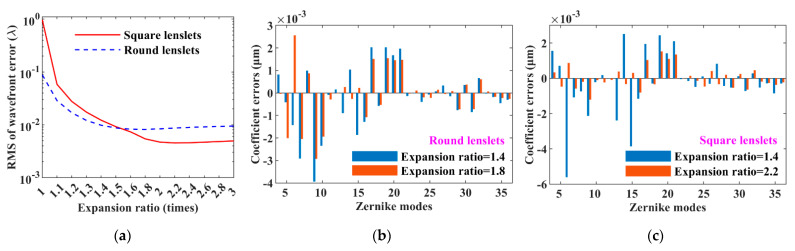
Effect of zero-padding on the performance of vSHWS. (**a**) RMS varying with expansion ratio; Zernike coefficient errors at optimized and non-optimized expansion ratios when using (**b**) round and (**c**) square lenslets, respectively.

The vSHWS can avoid the phenomena of overlapped spots and spot crossover between the adjacent sub-apertures caused by the excessive slope of the sub-wavefront, and thus has a high dynamic range. The zero-padding of the sub-aperture electric field *E*_0_ makes the centroid calculation more accurate. Figure 3a shows the RMS plots of wavefront errors varying with the expansion ratio. We can make a number of observations here: First, the zero-padding of *E*_0_ can significantly improve the performance, especially with the RMS being decreased by two orders of magnitude when using the square lenslets. Second, the vSHWS using the round lenslets performs better when the expansion ratio is less than ≈1.53, but the situation gets reversed when the expansion ratio is greater than ≈1.53, which confirms the assumption in Section 3.1. Third, the RMS plots tend to be flat when the expansion ratio is large enough, but it occurs more later when using the square lenslets. Lastly, the optimal expansion ratios are about 1.8 and 2.2 for using the round and square lenslets, respectively; the performance gain is very limited, but the computation burden is greatly increased when the expansion ratios continue to increase. Figure 3b,c shows respectively the Zernike coefficient errors at expansion ratios of 1.4 and 1.8 when using the round lenslets and also those at expansion ratios of 1.4 and 2.2 when using the square lenslets. By changing the expansion ratios from 1.4 to the optimal values, the changes of the Zernike coefficient errors of vSHWS with the round lenslets were less than those with the square lenslets. The Zernike coefficient errors in Figure 3c are smaller than those in Figure 3b at the corresponding optimal expansion ratios. Both of these results are consistent with the results in Figure 3a.

### 3.3. Number of Sub-Apertures

**Figure 4:**
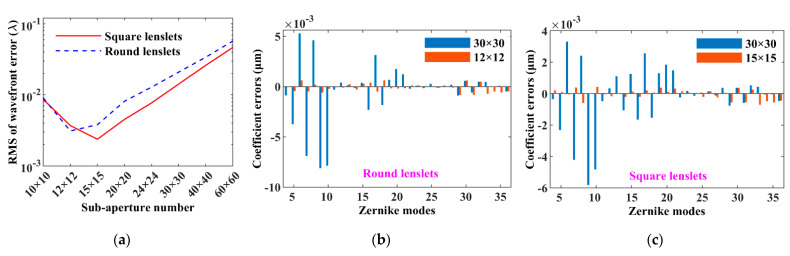
Effect of sub-aperture number on the performance of vSHWS. (**a**) RMS varying with sub-aperture number; comparisons of Zernike coefficient errors at non-optimized and optimized sub-aperture numbers when using (**b**) round and (**c**) square lenslets, respectively.

The RMSs of the wavefront errors varying with the sub-aperture number are shown in Figure 4a, and it can be seen that (1) the effects of the sub-aperture number on the performance of vSHWS are similar for using the round and square lenslets; (2) when the sub-aperture number is changed from small to large, the wavefront errors decrease first and then increase, which is consistent with the theoretic analysis in Section 2.2; (3) the optimal sub-aperture numbers are 12 × 12 and 15 × 15 for the round and square lenslets, respectively; (4) compared with the previous results (RMSs of 8.150 × 10^−3^ *λ* and 4.520 × 10^−3^ *λ* for the round and square lenslets, respectively) at a sub-aperture number of 20 × 20 shown in Figure 3a, the RMSs decrease at the optimal sub-aperture numbers for both the round (3.106 × 10^−3^ *λ*) and square (2.378 × 10^−3^ *λ*) lenslets. Figure 4b,c shows the Zernike coefficient errors at sub-aperture numbers of 30 × 30 and 12 × 12 when using the round lenslets, and those at sub-aperture numbers of 30 × 30 and 15 × 15 when using the square lenslets, respectively. The Zernike coefficient errors at low modes (less than about the 21th term) are significantly decreased at the optimized sub-aperture numbers.

### 3.4. Data Interpolation

**Figure 5:**
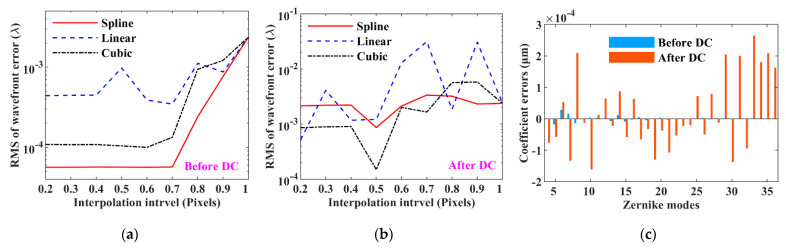
Effect of data interpolation on the performance of vSHWS. RMS varying with interpolation interval when performing interpolation (**a**) before and (**b**) after the DC; (**c**) comparisons of Zernike coefficient errors obtained before and after the DC.

Using three interpolation algorithms and the two interpolation sequences, the effect of changing the interpolation interval on the performance of vSHWS using the square lenslets was studied, and the results are shown in Figure 5. An interpolation interval of less than 0.2 pixels was not considered because the data amount was too large to be calculated and the obtained RMSs were low enough at this interval. Figure 5a,b shows the RMS plots of the wavefront errors varying with the interpolation interval when applying the three interpolation algorithms before and after the DC, respectively. When performing interpolation before the DC, we noted the following: (1) the RMS plots obtained by using the spline and cubic algorithms are similar, but that of the former is a little smaller; (2) compared with the previous optimal result (RMS of 2.378 × 10^−3^ *λ*) in Figure 4a, the spline algorithm reduces the RMS by two orders of magnitude to 5.671 × 10^−5^ *λ* at interpolation interval of 0.7 pixels; (3) the performance improvement of vSHWS is no longer obvious when the interpolation interval is less than 0.7 pixels. When performing interpolation after the DC, we noted the following: (1) the RMS plots obtained by using the spline and cubic algorithms are still similar but that obtained by using the latter is a little smaller, and their minimums appear at interpolation interval of 0.5 pixels and are 8.653 × 10^−4^ *λ* and 1.489 × 10^−4^ *λ*, respectively; (2) all the algorithms are unstable, especially the linear. If the interpolation is performed before the DC, the requirement of the interpolation interval will be low. Figure 5c shows the Zernike coefficient errors obtained by performing interpolation before and after the DC and by adopting the spline algorithm and an interpolation interval of 0.5 pixels. From it we can see that the Zernike coefficient errors obtained by performing data interpolation before the DC were much smaller compared with those obtained by performing data interpolation after the DC.

### 3.5. Results of Parameter Optimizations

**Figure 6:**
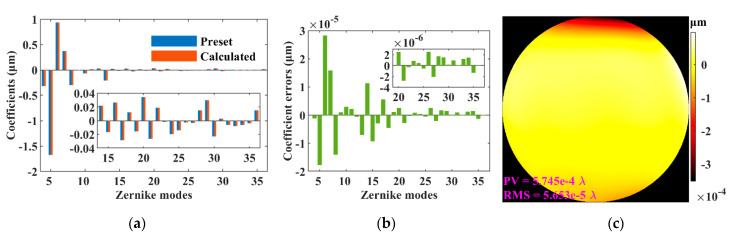
Results of vSHWS with optimizing parameters. (**a**) Zernike coefficients; (**b**) Zernike coefficient errors; (**c**) wavefront error.

So far, the optimal parameters of vSHWS were determined to be as follows: (1) a square shape for the virtual lenslets, (2) an expansion ratio of 2.2 times for the zero-padding of *E*_0_, (3) a sub-aperture number of 15 × 15, (4) performing interpolation before the DC, (5) using spline interpolation algorithm, and (6) an interpolation interval of 0.5 pixels. These optimal parameters were adopted to reconstruct *W*_P_. Figure 6a,b shows the Zernike coefficients of *W*_P_ and *W*_R_, respectively, as well as their errors. Comparing the results shown in Figure 2d and Figure 6b, it can be seen that the Zernike coefficient errors were reduced by four orders of magnitude. Figure 6c shows the wavefront error under the optimal configuration. Comparing the results shown in Figure 2e and Figure 6c, it can be seen that PV of the wavefront error was reduced from 3.858 *λ* to 5.745 × 10^−4^ *λ*, and RMS of the wavefront error was reduced from 0.971 *λ* to 5.653 × 10^−5^ *λ*. These results illustrate that the performance of vSHWS can be significantly improved by optimizing the parameters.

### 3.6. Anti-Noise Performance

**Figure 7:**
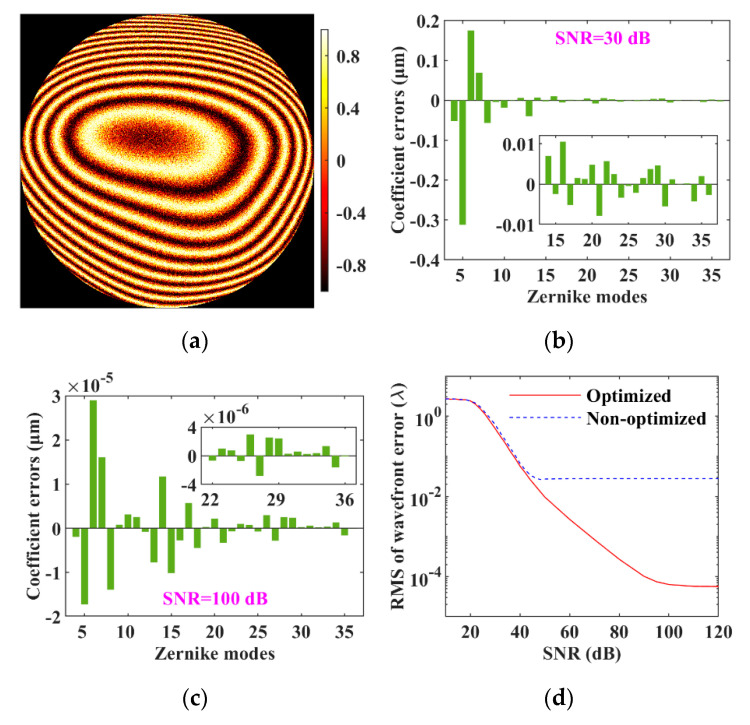
Anti-noise performance of vSHWS with and without parameter optimizations. (**a**) Real part of *E* generated by using a preset wavefront with a SNR of 30 dB; Zernike coefficient errors obtained by the optimized vSHWS at SNRs of (**b**) 30 dB and (**c**) 100 dB; (**d**) performance of the optimized and non-optimized vSHWS at different SNRs.

The effect of the parameter optimizations on the anti-noise performance of vSHWS was also investigated. Different white Gaussian noises were added to *W*_P_ to generate the wavefronts with different signal-to-noise ratios (SNRs), which were calculated in decibel units on 10·log10. These wavefronts were then reconstructed by using the non-optimized and optimized vSHWS, respectively. The non-optimized vSHWS was configured as follows: square virtual lenslets, sub-aperture number of 20 × 20, expansion ratio of 1.2 times for the zero-padding of *E*_0_, and no data interpolation. The real part of *E* with an SNR of 30 dB is shown in Figure 7a as an example, and it is seriously blurred. Figure 7b,c shows the Zernike coefficient errors obtained by using the optimized vSHWS at SNRs of 30 and 100 dB, respectively, and it can be seen that the Zernike coefficient errors are much smaller at SNR of 100 dB. The RMS plots of the wavefront errors varying with SNR are shown in Figure 7d. All the RMSs obtained by the optimized vSHWS were smaller than those obtained by the non-optimized vSHWS, illustrating that the anti-noise performance was improved by the parameter optimizations. The RMS plot tends to be flat from SNR of ≈45 dB for the non-optimized vSHWS, while it appears from SNR of ≈100 dB for the optimized vSHWS. In addition, at an SNR of 100 dB, the RMS (6.316 × 10^−5^ *λ*) obtained by the optimized vSHWS was three orders of magnitude smaller than that (2.770 × 10^−2^ *λ*) obtained by the non-optimized vSHWS. The two results illustrate that the optimized vSHWS gains more benefit at high SNR.

### 3.7. Clinical Human Ocular Aberrations

In order to avoid the obtained occasional results shown above when using only one set of data, 20 sets of clinical human ocular aberrations were also used to evaluate the performance of the optimized vSHWS. The data were clinically collected with an aberrometer (KR-1W, Topcon Corp, Tokyo, Japan) at a pupil diameter of 6 mm from 20 patients at the Eye Hospital of Wenzhou Medical University. They were diagnosed by experienced physicians to two groups: 10 normal eye patients with an age range from 6 to 75 years with a mean age of 38 years, and 10 diseased eye patients with an age range from 19 to 58 years with a mean age of 37.8 years. Data processing was performed the same way as previously: First, the Zernike coefficients were used to generate the preset *E*, and then the optimized vSHWS was used to obtain the Zernike coefficients from the preset *E*. The Zernike coefficient errors, RMS, and PV of the wavefront errors between *W*_R_ and *W*_P_ for every eye were recorded, and the statistical Zernike coefficient errors for all normal and all diseased eyes were finally obtained.

**Figure 8:**
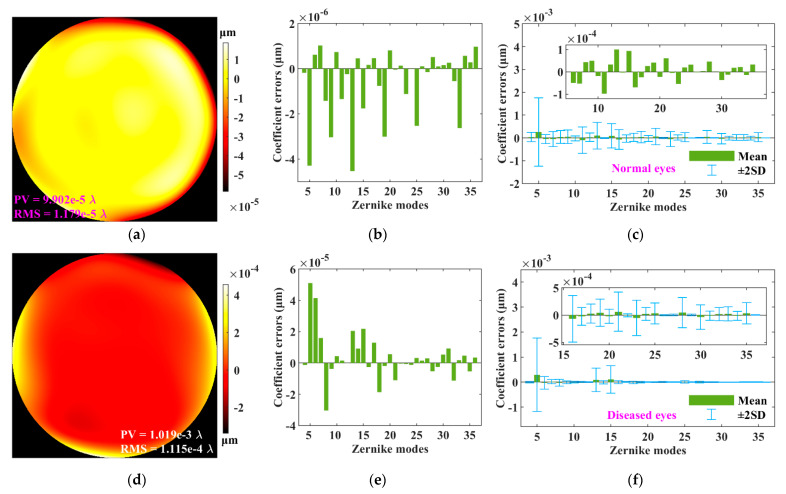
Performance of optimized vSHWS used for clinical ocular aberrations. (**a**) Wavefront error and (**b**) coefficient errors obtained with a set of normal eye’s aberrations; (**c**) statistical coefficient errors for 10 normal eyes; (**d**) wavefront error and (**e**) coefficient errors obtained with a set of diseased eye’s aberrations; (**f**) statistical coefficient errors for 10 diseased eyes.

As an example, Figure 8a,b shows the wavefront error and the Zernike coefficient errors when using the data from a normal eye (Patient 5 in Table 1), respectively. Figure 8c shows the statistical Zernike coefficient errors with a form of mean ± 2SDs (standard deviations) for all the normal eyes’ aberrations. Similarly, the same terms are shown in Figure 8d,e for a diseased eye (Patient 5 in Table 1) and in Figure 8f for all the diseased eyes. From Figure 8c,f, it can be found that all the mean ± 2SDs’ absolute values of the Zernike coefficient errors, except for the 5th term (i.e., the defocus), are less than 7 × 10^−4^ μm for both the normal and diseased eyes, although these values (on the order of 10^−4^ μm) are increased by two orders of magnitude compared to the result (on the order of 10^−6^ μm) in Figure 6b, but still small. The worst from the 5th term is less than 2 × 10^−3^ μm for both the normal and diseased eyes, and it is also not too large. The RMSs and PVs of the obtained wavefront errors for every patient are listed in Table 1. Most of the RMSs are on the order of 10^−5^ *λ* for the normal eye and 10^−4^ *λ* for the diseased eye, and most of the PVs are on the order of 10^−4^ *λ* for all eyes. The worst RMS and PV (Patient 2 of normal eye in Table 1) are still on the order of 10^−3^ *λ* and 10^−2^ *λ*, respectively. Therefore, the results obtained by using clinical data with 20 samples demonstrated that as a whole, the optimized vSHWS has a high performance for wavefront sensing.

## 4. Discussion

The complex electric field *E* of the beam at the pupil needs to be obtained first by using a designated method for the task, such as interferometry, and then vSHWS can be used to obtain wavefront aberrations. Currently, vSHWS is usually used for CGWS. We saw that vSHWS is more stable against the phase singularities compared to the methods for performing phase unwrapping directly [14]. Therefore, their combination, i.e., CG-vSHWS, is suitable for the aberration measurements of inhomogeneous biological specimens. The main disadvantage of CG-vSHWS is that interferometry complicates the system and alignment, but it is no longer a problem when it is combined with adaptive optics optical coherence tomography.

There are many algorithms for centroid calculation, such as calculating first-order moment, windowing, and thresholding [29,30], etc. The first one was used in this work, due to its simple principle and fast calculation speed. However, this algorithm becomes unstable as the noise becomes strong, and a more stable algorithm should be used in practice. Wavefront reconstruction algorithms can be classified into zonal and modal reconstructions [7], and the Zernike reconstruction used in this work is a kind of the latter. In addition, there are also Taylor reconstruction, Fourier reconstruction, and iterative Fourier reconstruction to be used.

From Figure 3a, we can see that the zero-padding of *E*_0_ can significantly improve the performance of vSHWS. The optimal RMS (4.520 × 10^−3^ *λ*) of the wavefront error when using the square lenslets is smaller than that (8.150 × 10^−3^ *λ*) when using the round lenslets because the v-LA composed of the square lenslets has a higher fill factor and thus uses more *E* information. However, the round lenslets should still be considered a candidate that can be used for the following occasions: (1) the performance of vSHWS using the round lenslets is better when a low expansion ratio is required to reduce the computation burden; (2) there is not much difference between the RMSs of the wavefront errors obtained by using the round and square lenslets at their corresponding optimal expansion ratios, but the calculation burden of the latter is increased.

Data interpolation can improve data density, which is beneficial for improving the accuracy of centroid calculation and thus improve the sensitivity of wavefront sensing. The results from Figure 5 indicate that a proper interpolation strategy can significantly improve the performance of vSHWS. We can see from Figure 5b that the RMSs of the wavefront errors obtained at some interpolation intervals, such as 0.6, 0.8, and 0.9 pixels, are higher than those obtained without data interpolation. The reason may be that an improper interpolation interval causes too much original data loss during resampling. Therefore, the interpolation intervals that should be used are the ones where we can obtain as much original data as possible, and those satisfying the Nyquist–Shannon sampling theorem are recommended, especially when performing interpolation after the DC. Comparing Figure 5a,b, we can see that performing interpolation before the DC is a better choice for all the interpolation algorithms. However, not only the RMS of the wavefront error but also the computation burden should be considered in practice, especially for real-time aberration measurement. Because the time-consuming fast Fourier transform is required for the DC, the computation burden of performing interpolation after the DC is less than that before the DC. On the premise that the accuracy of wavefront sensing meets the application requirement, it is recommended to perform interpolation after the DC.

For SHWS, the focused spot position is confined by the sub-aperture size [31], and there is a trade-off between the dynamic range and the sensitivity. The focal length *F* of the lenslet is an important parameter for SHWS because it is related to both the dynamic range and the sensitivity [16]. For vSHWS, both the dynamic range and the sensitivity can be increased because *E*_0_ is processed separately, and the focused spot centroid can be calculated no matter how the wavefront is distorted. Equation (5) shows that the centroid displacement Δ*s* is related to *F*, but *F* is eliminated when calculating the wavefront slope *θ*. Therefore, *F* of the virtual lenslet may not affect the performance of vSHWS. In order to verify this assumption, *W*_P_ was reconstructed by the optimized vSHWS at different *F* from 5 to 150 mm. The result shows that the RMS of the wavefront error almost did not change (the maximal change was 9.932 × 10^−13^ *λ*), and the above assumption was verified to be accurate.

The data processing time of vSHWS was longer than that of SHWS because it calculates the light propagation and performs the zero-padding and the data interpolation. However, these calculations are easy to complete for a well-configured computer. We ran the programs of an optimized vSHWS and a real SHWS to process the same set of wavefront aberrations for 10 times on a same PC; the runtimes were recorded, and the averages were obtained to be 3.367 s and 1.621 s, respectively. We can see that the former is about twice the latter, but they are still on the same order of magnitude. Furthermore, the processing time of vSHWS can be reduced by improving the processing algorithms and the PC’s performance, and in this way it can meet the requirements of most applications.

For real SHWS, an incident beam is divided into multiple sub-beams and then is focused on a 2D detector by an LA. For vSHWS, the above process used is a Fresnel diffraction approximation, which is a general method for diffraction calculation and is widely used in physical optics. Therefore, the computation error indeed exists in vSHWS, but it does not have a big impact on the result.

The method for obtaining the optimal parameters is the focus of this work. In principle, the obtained optimal parameters in this work cannot be used as a general standard of vSHWS, and they should be found out according to detailed requirement and special application. Fortunately, the study in Section 3.7 demonstrated that the optimized vSHWS obtained with a set of ocular aberrations also worked well when it was used for other aberrations directly. Therefore, some results and conclusions in this work may be adopted by other works if their measurement conditions are similar to our conditions.

## 5. Conclusions

The working process of vSHWS was described in detail, and some optimization methods were used to improve the performance. The wavefront to be measured was preset by using a set of Zernike coefficients of normal human ocular aberrations, and the procedure and methods for reconstructing the preset wavefront using vSHWS were shown. The effects of the lenslet shape, the *E*_0_ zero-padding, the sub-aperture number, and the data interpolation of *E*_0_ or *I*_F_, on the performance of vSHWS were studied. The Zernike coefficient errors and the RMS of the wavefront error between the reconstructed and preset wavefronts were used to evaluate the performance, and the optimal configuration of vSHWS was finally obtained. By comparing the reconstructed wavefronts obtained by the optimized and non-optimized vSHWSs, it was found that the wavefront sensing accuracy and the anti-noise performance could be significantly improved by the parameter optimizations; more specifically, the RMS of wavefront error indicating the sensing accuracy was reduced from 0.971 *λ* to 5.653 × 10^−5^ *λ* in this work. The performance of the optimized vSHWS was also verified with 20 sets of clinical human ocular aberrations including normal and diseased eyes.

## Figures and Tables

**Table 1:** RMSs and PVs of the wavefront errors obtained by using clinical human ocular aberrations (data for calculations Appendix A).

Type	Parameter	Patient									
		1	2	3	4	5	6	7	8	9	10
Normal eye	PV (× 10^−4^ λ)	4.591	399.364	1.354	11.789	0.990	4.477	3.985	3.540	1.892	7.085
	RMS (× 10^−5^ λ)	5.561	450.722	1.159	16.702	1.179	6.708	2.677	5.187	1.317	5.521
Diseased eye	PV (× 10^−4^ λ)	2.241	9.286	193.745	6.921	10.192	19.965	5.661	7.646	3.133	4.631
	RMS (× 10^−4^ λ)	0.160	1.641	36.144	0.836	1.115	1.585	0.416	1.730	0.507	0.938

## Data Availability

The normal human ocular aberrations used in Section 2.1 are available at Ref. [24]. The clinical human ocular aberrations used in Section 3.7 are listed in Table A1 and Table A2.

## Appendix A

The clinical human ocular aberrations used in Section 3.7 are listed in Table A1 and Table A2.

**Table A1:** The Zernike aberration coefficients of 10 normal human eyes (in μm).

Zernike Modes	Patient									
	1	2	3	4	5	6	7	8	9	10
4	−0.1973	−0.1600	−0.0925	0.1798	−0.0524	0.8857	0.1599	0.0292	−0.3909	0.9966
5	−1.8435	9.6149	0.9575	2.9316	−0.1412	2.6880	−1.1025	1.1807	0.6760	−0.4776
6	−1.5900	−0.5373	0.0105	−0.9978	0.0445	−0.6290	1.0174	−2.0036	0.1262	−0.2747
7	0.0666	−0.3082	−0.0627	−0.0836	−0.1233	−0.0887	−0.2842	−0.0310	0.1864	−0.6684
8	0.2207	0.1601	0.0376	−0.0428	0.1987	−0.1467	−0.0487	0.2097	0.1675	−0.1657
9	0.0768	−0.0495	0.0191	−0.0684	−0.0795	−0.1524	0.1163	0.0440	−0.0941	−0.0837
10	−0.1374	0.2678	−0.0243	−0.0348	0.0857	0.3965	−0.1188	−0.0619	0.0705	−0.8523
11	0.0739	−0.4793	0.0419	−0.0052	−0.0557	0.0391	−0.1254	−0.0428	0.0166	−0.1590
12	−0.0330	0.3016	−0.0630	0.0507	0.0398	0.0923	0.0085	0.1183	0.0001	0.0238
13	0.1209	0.0253	0.3635	0.2708	0.3113	−0.3632	0.1269	0.1255	−0.1646	0.2152
14	−0.0378	0.1148	−0.0328	−0.1305	−0.0630	0.1100	−0.1113	−0.0117	0.0465	−0.0295
15	−0.0053	0.0940	0.0987	0.0909	0.0644	−0.1782	0.1866	−0.0157	−0.0098	0.3866
16	0.0214	−0.4494	−0.0233	0.0491	−0.0205	0.0425	0.0358	−0.0563	0.0180	0.2729
17	0.0160	0.0497	0.0377	0.1049	−0.0002	0.0995	0.0007	−0.0132	−0.0167	0.0658
18	−0.0338	−0.0204	−0.0215	0.0105	−0.0084	−0.0123	0.0052	0.0060	−0.0095	0.0997
19	−0.0013	0.0939	0.0191	0.0309	−0.0010	−0.0498	−0.0106	−0.0163	−0.0077	−0.1261
20	0.0141	−0.1724	−0.0289	−0.0166	−0.0058	−0.1283	0.0419	0.0664	−0.0355	0.1476
21	−0.0205	0.0664	−0.0302	0.0104	0.0582	0.1305	−0.0508	0.0547	−0.0245	−0.0985
22	−0.0070	−0.2003	−0.0203	−0.0140	0.0004	0.0021	−0.0366	−0.0541	−0.0231	−0.0927
23	−0.0290	−0.3285	0.0433	−0.0046	0.0137	−0.0224	0.0303	0.0133	−0.0107	−0.0202
24	0.0070	0.0434	−0.0355	0.0134	−0.0024	0.0041	0.0001	−0.0058	0.0257	−0.0754
25	−0.0390	−0.0452	0.1052	0.0086	0.0070	−0.0213	0.0166	−0.0020	0.0434	0.0551
26	0.0480	0.0865	−0.0216	0.0108	0.0029	−0.0102	−0.0553	0.0495	0.0060	0.0301
27	−0.0222	−0.0008	0.0349	−0.0285	0.0018	0.0488	0.0112	−0.0256	−0.0002	−0.0885
28	−0.0005	0.3029	0.0144	0.0097	−0.0191	−0.0563	−0.043	0.0361	0.0107	−0.0521
29	0.0367	−0.0572	0.0100	−0.0239	−0.0282	0.0178	−0.0054	0.0332	0.0576	−0.0101
20	−0.0202	−0.2556	−0.0118	−0.0128	0.0024	0.0177	0.0011	0.0223	−0.0085	−0.0687
31	−0.0030	−0.0619	0.0122	−0.0099	−0.0012	−0.0352	−0.0189	−0.0041	0.0099	0.0154
32	−0.0158	−0.0431	−0.0020	0.0289	0.0050	0.0273	0.0239	0.0051	0.0041	0.0500
33	0.0002	0.0559	0.0006	0.0083	0.0116	0.0221	0.0090	0.0011	−0.0061	−0.0026
34	−0.0121	0.0354	−0.0081	−0.0128	−0.0086	0.0322	0.0106	−0.0126	0.0230	−0.0057
35	0.0190	0.2953	−0.0097	−0.0513	−0.0141	−0.0776	0.0008	−0.0060	0.0120	0.0434
36	0.0119	0.0780	0.0219	−0.0089	0.0080	0.1336	0.0302	0.0200	−0.0255	0.0424

**Table A2:** The Zernike aberration coefficients of 10 diseased human eyes (in μm).

Zernike Modes	Patient									
	1	2	3	4	5	6	7	8	9	10
4	0.1626	−0.2911	0.1316	−0.1530	−0.2043	0.3131	0.3247	−0.8298	−0.0350	−0.4166
5	−0.3104	3.8403	10.2138	2.4758	2.4199	1.9592	−0.2767	4.0751	2.5128	3.2984
6	0.2264	0.5834	−0.5318	−0.7658	1.0672	0.1734	0.2948	0.5710	−0.4314	−0.2774
7	−0.5651	0.1585	0.1009	0.1373	0.0926	0.5468	−0.1791	−0.2911	−0.0166	−0.2369
8	0.2698	0.2721	−0.2064	−0.2573	−0.4067	0.1804	0.0005	−0.3212	0.1435	−0.0581
9	−0.1277	0.0379	−0.0875	−0.0820	−0.1160	0.3371	−0.1922	−0.0093	−0.0408	0.0255
10	0.3687	−0.3038	0.1127	0.1048	0.0933	0.0274	−0.0387	−0.0587	−0.0677	0.0936
11	−0.1098	0.0684	0.0131	0.0163	0.0930	0.2226	0.1782	−0.0780	−0.0180	−0.0257
12	−0.0485	−0.0324	−0.0197	−0.0083	0.0772	0.1399	0.0439	−0.0078	−0.0066	0.0186
13	−0.1248	−0.1533	0.2039	0.0569	0.2442	0.1056	0.3033	−0.7340	−0.2086	−0.0657
14	0.0312	−0.0659	0.0166	−0.0347	−0.0636	0.0188	0.1143	0.1575	−0.0082	0.1106
15	−0.0591	−0.0734	−0.0129	0.0903	0.0449	0.0306	−0.0804	0.0030	0.0363	−0.0299
16	−0.1921	0.0772	−0.0319	0.0170	−0.0583	0.0887	−0.0617	0.0597	0.0917	−0.0281
17	0.1646	−0.0145	−0.0165	0.0316	0.0618	0.0217	0.0466	0.0686	0.0149	0.1507
18	−0.0372	−0.0011	−0.0132	−0.0253	0.0306	0.0747	−0.0790	−0.0316	0.0397	−0.0238
19	−0.0372	0.1007	−0.0029	0.0420	−0.0439	0.0981	0.0151	−0.0077	−0.0001	−0.0321
20	−0.0539	0.0732	−0.0178	−0.0540	−0.0792	0.0467	0.0350	0.0001	−0.0235	0.0058
21	0.0263	0.0578	−0.0183	−0.0273	−0.0577	0.0110	0.0299	0.0988	−0.0406	−0.0371
22	−0.0177	0.1391	0.0002	−0.0153	−0.0644	0.0944	0.0121	−0.0036	−0.0330	−0.0094
23	0.0056	−0.0280	0.0035	−0.0220	0.0117	0.0689	−0.0539	0.0194	−0.0035	−0.0134
24	0.0139	0.0140	−0.0027	−0.0299	0.0264	0.0565	0.0174	0.0157	−0.0363	0.0166
25	−0.0487	0.0435	0.0081	−0.0118	0.0375	0.1115	0.0160	0.0042	−0.0502	−0.0346
26	−0.0123	−0.0393	0.0153	−0.0014	−0.1286	−0.0318	−0.0552	−0.0137	0.0297	0.0148
27	−0.0117	−0.0372	−0.0034	0.0121	0.0100	0.0245	0.0399	−0.0083	−0.0033	−0.0169
28	0.0745	−0.2031	0.0139	−0.0151	0.0137	0.0271	−0.0326	0.0377	−0.0175	0.0123
29	−0.0147	0.0378	−0.0198	0.0032	0.0774	0.2117	−0.0379	0.0303	0.0905	−0.0405
20	0.0319	−0.0204	0.0050	0.0031	0.0396	−0.0175	−0.0168	−0.0118	−0.0307	−0.0168
31	−0.0349	−0.0280	0.0059	−0.0101	−0.0376	0.0827	−0.0061	−0.0273	0.0048	−0.0365
32	0.0237	−0.0032	0.0072	0.0073	0.0868	0.0159	−0.0143	0.0305	0.0140	0.0239
33	0.0187	0.0053	−0.0070	−0.0077	−0.0411	−0.0018	0.0500	−0.0076	0.0079	−0.0122
34	−0.0043	−0.0098	0.0023	0.0080	−0.0220	−0.0298	0.0257	−0.0080	0.0014	0.0075
35	0.0115	0.0303	0.0201	0.0041	−0.0132	0.0582	−0.0543	−0.0066	−0.0010	0.0294
36	0.0436	−0.0069	0.0101	0.0030	0.1714	0.0186	0.0044	−0.0035	0.0032	0.0189
